# Antioxidant Properties and Digestive Enzyme Inhibitory Activity of the Aqueous Extract from Leafy Stems of *Cissus polyantha*

**DOI:** 10.1155/2019/7384532

**Published:** 2019-11-16

**Authors:** Mahamad Abba Talba, David Miaffo, Sylviane Laure Poualeu Kamani, Albert Kamanyi, Sylvie Léa Wansi

**Affiliations:** ^1^Department of Biological Sciences, Faculty of Sciences, University of Maroua, Far North Region, Maroua, Cameroon; ^2^Department of Life and Earth Sciences, Higher Teachers' Training College, University of Maroua, Far North Region, Maroua, Cameroon; ^3^Department of Animal Biology, Faculty of Sciences, University of Dschang, West Region, Dschang, Cameroon

## Abstract

*Cissus polyantha* (Vitaceae) is a plant used in Cameroonian traditional medicine for the treatment of diabetes. The aims of this study were to evaluate the in vitro antioxidant and antidiabetic activities of the aqueous extract of *Cissus polyantha* leafy stems. The enzyme inhibitory activity was determined in vitro on *α*-amylase and *α*-glucosidase enzymes, followed by confirmative study in vivo on normal rats (oral starch and sucrose tolerance tests at doses of 111, 222, and 444 mg/kg). The ferric reducing antioxidant power and the 2,2-diphenyl-1-picrylhydrazyl (DPPH) antiradical activity of the extract were examined to evaluate the antioxidant potential of the extract. The total content of phenols, flavonoids, and tannins of the extract were also determined. The results showed an inhibitory effect of the extract on the *α*-amylase and *α*-glucosidase activities with IC_50_ values of 216.14 and 182.40 *μ*g/mL, respectively. The extract at doses of 222 and 444 mg/kg induced a significant decrease in postprandial glycaemia during the starch and sucrose tolerance tests. A remarkable antiradical activity of the extract was obtained although lower than that of the standard product. The aqueous extract of leafy stems of *Cissus polyantha* has an interesting inhibitory activity on the *α*-amylase and *α*-glucosidase enzymes, as well as an antioxidant potential, thus validating its use in traditional medicine for the treatment of diabetes mellitus and its complications.

## 1. Introduction

Type 2 diabetes mellitus is a metabolic disease characterized by increasing in insulin resistance and decreased beta cell function and chronic hyperglycemia. It is responsible for the nonregulation of postprandial hyperglycemia which is a major factor of the cardiovascular complications [1]. One of the control pathways for postprandial hyperglycemia in diabetic patients is the inhibition of the activity of *α*-amylase and *α*-glucosidase enzymes [2]. Despite conventional treatments, the incidence and prevalence of diabetes are increasing all over the world and make it a major public health problem [3].

Oxidative stress is potentially involved in many diseases including diabetes, as a triggering factor or associated complications during its evolution [4]. Antioxidants are naturally present in our diet but in insufficient quantity to meet the body's needs. The use of synthetic antioxidant molecules is currently being questioned because of the potential toxicological risks.

The natural plant extract is used as the alternative therapeutic agents. Several plants have already shown their antioxidant and/or inhibitory potentials on the activity of *α*-amylase and *α*-glucosidase enzymes, such as *Retama raetam* [5], *Picralima nitida* [6], and *Combretum molle* [7]. *Cissus polyantha* (Vitaceae) is used in northern Nigeria as an antimicrobial and analgesic [8], in Liberia for the treatment of conjunctivitis [9], and in Cameroon for cure diarrheal diseases and diabetes. Qualitative phytochemical studies have reported that *C. polyantha* leaves contains quinoids, sterols, terpenes, saponins, phenols, tannins, and flavonoids [10]. The present study aims to quantify the polyphenols, flavonoids, and tannins in the aqueous extract from leafy stems of *Cissus polyantha*, to determine its antioxidant potential and to evaluate its inhibitory properties on the *α*-amylase and *α*-glucosidase activities.

## 2. Materials and Methods

### 2.1. Chemicals and Drugs

Acarbose, *α*-glucosidase, *α*-amylase, 3,5-dinitrosalicylic acid, p-nitrophenyl-D-glucoside (pNPG), and DPPH were purchased from Sigma-Aldrich (St. Louis, USA). Starch and sucrose were purchased from Edu-Lab Biology Kit (Bexwell, UK). All others chemicals and drugs used were of analytical grade available commercially.

### 2.2. Animals

Male Wistar rats weighing between 220 and 250 g and 8 to 12 weeks of age were provided by the animal house of the Department of Biological Sciences at the University of Ngaoundere (Cameroon). These animals were housed in plastic cages with free access to water and food at room temperature and in a natural environment. All rats were acclimated for 7 days before starting each test. The animal protocol was approved by the Institutional Animal Ethics Committee (IAEC) before starting the study.

### 2.3. Plant Material and Preparation of Extract

The leafy stems of *Cissus polyantha* were collected in September 2017 from Mémé, Far North Region-Cameroon, authenticated in the National Herbarium of Yaoundé-Cameroon where the voucher specimen was deposited under the number 44346/NHC. The leafy stems were dried and ground to obtain a fine powder.

One-hundred gram (100 g) of the dried powdered plant was infused in 500 mL of distilled water for 30 minutes. After filtration and evaporation in an oven, 18.8 g of the crude extract of *C. polyantha* leafy stems was obtained.

### 2.4. Total Phenol, Flavonoid, and Tannin Content

Total phenols of aqueous extract of *C. polyantha* were estimated using the Folin–Ciocalteu method. The absorbance was measured at 640 nm using a spectrophotometer. The concentration of total phenols was calculated using a calibrated curve of gallic acid. The results were expressed as mg GAE/g dry wt. (GAE = gallic acid equivalent).

Total flavonoids were estimated using the aluminium colorimetric method of Zhishen et al. [11] using quercetin as standard. The concentration of total flavonoids was calculated using a calibrated curve of standard quercetin. The results were expressed as mg QAE/g dry wt. (QAE = quercetin equivalent).

Tannin content was measured by the method of Bainbridge et al. [12] using cathechin as standard. The concentration of total tannins was calculated using a calibrated curve of cathechin. The results were expressed as mg CAE/g dry wt. (CAE = catechin equivalent).

All the analysis were carried out in triplicate.

### 2.5. In Vitro Enzyme Inhibition Study

#### 2.5.1. In Vitro *α*-Amylase Inhibition Study

The in vitro study of the inhibition of *α*-amylase enzyme was carried out according to the method described by Apostolidis et al. [13]. Twenty-five microliters (25 *μ*L) of extract or acarbose (1, 3, 10, 30, 100 and 300 *μ*g/mL) was added to 25 *μ*L of buffer phosphate (20 mM, pH 6.9), containing the solution of *α*-amylase (0.5 mg/mL), and the mixture was incubated at 25°C for 10 minutes. Subsequently, 25 *μ*L of 0.5% starch solution in phosphate buffer (20 mM, pH 6.9) was added to each tube to begin the reaction. The reaction mixtures were then incubated at 25°C for 10 minutes. The reaction was stopped by addition of 50 *μ*L of 3,5-dinitrosalicylic acid (96 mM) in each tube. The microplate was then incubated in a water bath for 5 minutes and then cooled to room temperature. Absorbance (A) was measured at 540 nm using a spectrophotometer. The inhibition percentage was calculated as follows: *I*% = (Ac − As)/Ac × 100, where Ac is the absorbance of the control and As is the absorbance of the sample. Distilled water was used as control and represents 100% enzymatic activity. The concentration of the extract necessary to inhibit the activity of the enzyme by 50% (IC_50_) was determined from the regression curve.

#### 2.5.2. In Vitro *α*-Glucosidase Inhibition Study

The in vitro *α*-glucosidase inhibition study was carried out according to the method described by Kim et al. [14]. In fact, 50 *μ*L of the solution of the extract or acarbose (1, 3, 10, 30, 100, and 300 *μ*g/mL) was introduced into 100 *μ*L of tris buffer (20 mM, pH 6.8) containing 100 *μ*L of the solution of *α*-glucosidase (0.01 mg/mL) and preincubated at 25°C for 10 min. After preincubation, 50 *μ*L of 5 mM pNDG (p-nitrophenyl-*α*-D glucopyranoside) was added to each tube to begin the reaction. The mixture was then incubated at 37°C for 15 min. The reaction was stopped after the addition of 2 mL of 500 mM Na_2_CO_3_ to each tube. Absorbance of the solution was read at 400 nm using a spectrophotometer. The inhibition percentage (*I*%) and the mean inhibition concentration (IC_50_) of the extract were calculated as in the case of *α*-amylase.

### 2.6. Oral Starch and Sucrose Tolerance Tests

Twenty-five (25) rats were fasted for 16 hours and randomly divided into 5 groups of 5 rats each as follows [15]:

- Group 1 (control) received 10 mL/kg of distilled water
- Group 2 (standard) received acarbose solution at dose of 10 mg/kg b.w.
- Group 3 received the extract at the dose of 111 mg/kg b.w.
- Group 4 received the extract at the dose of 222 mg/kg b.w.
- Group 5 received the extract at the dose of 444 mg/kg b.w.

Ten minutes after the administration of different treatments, the rats were given orally 4 g/kg b.w. of sucrose or 3 g/kg b.w. of starch. The blood glucose level was measured before the treatment and at the 30th, 60th, 90^th^, and 120th minutes after the hyperglycemia induction.

### 2.7. In Vitro Antioxidant Tests

#### 2.7.1. Ferric-Reducing Antioxidant Power

The reducing capacity of Fe3+ to Fe2+ by plant extract was determined by the method of Chaouche et al. [16]. 200 *μ*L of extract at different concentrations (0 to 800 *μ*g/mL), 500 *μ*L of phosphate buffer (2 mM), and 500 *μ*L of a solution of K3Fe (CN) 6 (1%) were mixed. After incubation at 50°C for 20 min, 500 *μ*L of trichloroacetic acid (10%) was added to stop the reaction. After that, 1.7 mL of distilled water and 200 *μ*L of 0.1% (w/v) FeCl_3_ were added to the mixture. Absorbance was read against blank at 700 nm. The results were used to calculate the effective concentration (EC_50_) from the linear regression curve (DO = *f* (*C*)) and compared to those of ascorbic acid used as a positive control. All tests and analyses were carried out in triplicate.

#### 2.7.2. DPPH Antiradical Activity

The DPPH (2,2-diphényl-1-picrylhydrazyl) antiradical effect of *C. polyantha* extract was determined by the method of Sayyed et al. [17]. A DPPH solution was prepared in ethanol (0.5 mM). Then, 2 mL of this solution was introduced into a test tube containing 3 mL of extract at different concentrations (0 to 800 *μ*g/mL). The mixture was stirred well for 5 min at room temperature (25°C). For the control tube, distilled water was used in place of the extract. Butylhydroxyanisole (BHA) was used as positive control. Absorbance was read at 517 nm. The antioxidant activity of the extract was expressed as percent inhibition (%) according to the following equation: inhibition (%) = (Ac − As)/Ac × 100, where Ac is the control absorbance and As is the absorbance of the sample. The concentration of the plant extract and the positive control required to inhibit 50% of DPPH radicals (IC_50_) was calculated.

### 2.8. Statistical Analysis

The results were expressed as mean ± standard error of the mean (SEM). Data obtained were analyzed and means were compared using one-way or two-way ANOVA followed by Tukey and Bonferroni tests, respectively using Graph Pad Prism Version 5.03. IC_50_ and EC_50_ values were calculated using nonlinear regression analysis. *p* < 0.05 was considered to be statistically significant.

## 3. Results

### 3.1. Total Phenol, Flavonoid, and Tannin Contents

Table 1 shows that the aqueous extract of the leafy stems of *Cissus polyantha* contains phenols, flavonoids, and tannins. The amount of total phenols in the extracts was determined using gallic acid (standard curve equation *y* = 2.493*x* − 0.039, *R*^2^ = 0.991) as standard. The results indicated that the aqueous extract content was 167.89 ± 0.1 mg GAE/g dry wt. of total phenols. The flavonoid content (7.54 ± 0.15 mg QE/g dry wt.) was determined using quercetin as standard (standard curve equation: *y* = 25.85*x* + 0.028, *R*^2^ = 0.980). The tannin content (0.82 ± 0.5 mg CE/g dry wt.) was evaluated using catechin as standard (standard curve equation *y* = 1.079*x* + 0.052, *R*^2^ = 0.980). These results revealed that the aqueous extract of *Cissus polyantha* contains phenols, flavonoids, and tannins at varying proportions.

Values are means of three analyses of the extract ± standard deviation (*n* = 3). GAE: gallic acid equivalent; QE: quercetin equivalent; CE: catechin equivalent; dry wt.: dry weight.

### 3.2. In Vitro *α*-Amylase Inhibition Activity

**Figure 1:**
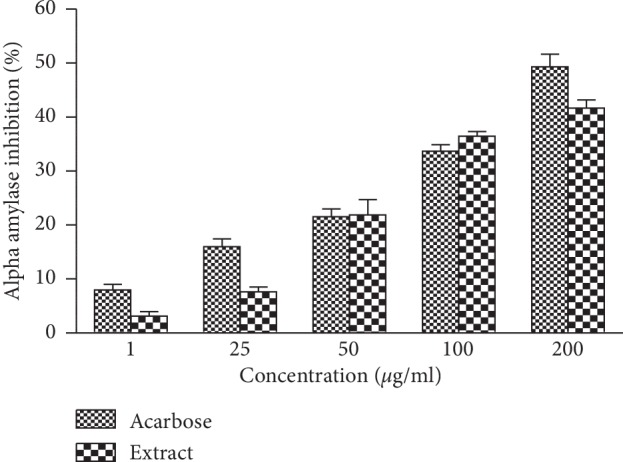
Inhibitory activity of aqueous extract of *Cissus polyantha* leafy stems on *α*-amylases. Values are expressed as mean ± SE. SE: standard error.

The results revealed an increased concentration dependency on the percentage inhibition of *α*-amylase enzyme (Figure 1). Indeed, acarbose has shown a significant inhibitory potential with inhibition percentages ranging between 7.98% and 49.30%, while the extract showed an inhibitory activity of 3.12% to 41.66% corresponding to concentrations ranging from 1 to 200 *μ*g/mL. The IC_50_ values of extract and acarbose were 195.07 ± 1.44 and 216.14 ± 0.83 *μ*g/mL, respectively.

### 3.3. In Vitro *α*-Glucosidase Inhibition Activity

**Figure 2:**
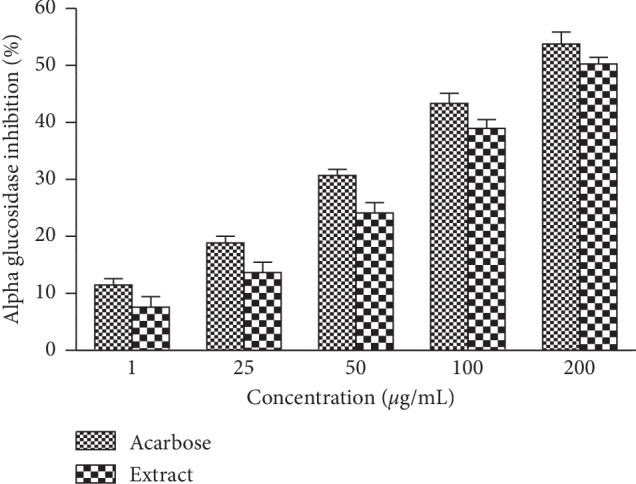
Inhibitory activity of aqueous extract of *Cissus polyantha* leafy stems on *α*-glucosidases. Values are expressed as mean ± SE. SE: standard error.

Figure 2 shows the inhibition percentage of the *α*-glucosidase enzyme as a function of acarbose and extract concentrations. Acarbose had a strong *α*-glucosidase inhibitory activity, which varies from 11.48% to 53.77% for concentrations between 1 and 200 *μ*g/mL. The IC_50_ value was 163.39 ± 1.22 *μ*g/mL. Similarly, leafy stem extract had significant inhibitory potential on *α*-glucosidase activity. This inhibition varies from 7.55% to 50.29% for concentrations ranging from 1 to 200 *μ*g/mL. Its IC_50_ value was 182.40 ± 0.54 *μ*g/mL.

### 3.4. Effects of the Aqueous Extract of *C. polyantha* on Oral Starch Tolerance Test

**Figure 3:**
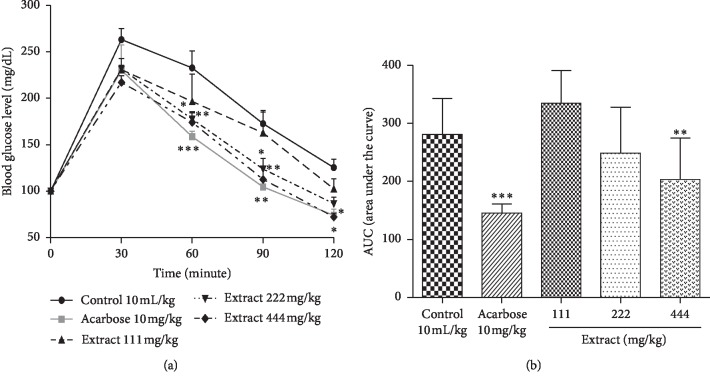
Effect of the aqueous extract of *Cissus polyantha* leafy stems on postprandial blood glucose level (a) and area under the curve (AUC) (b) after starch loading in normal rat. Values are expressed as mean ± SE of 5 rats in each group. ^*∗*^*p* < 0.05; ^*∗∗*^*p* < 0.01; ^*∗∗∗*^*p* < 0.001 compared to control. SE: standard error.

Figure 3 shows the results of the starch tolerance test in rats in temporary hyperglycemia treated with the aqueous extract of *C. polyantha.* In fact, the blood glucose of all the animals increased at the 30th minute and then gradually decreases until the end of the experiment. However, the blood glucose levels of animals treated with acarbose and the different doses of extract remained lower than those of rats in the control group.

When compared with animals of the control group, acarbose brought about significant reduction in the blood glucose levels of approximately 73.90% (*p* < 0.001), 68.11% (*p* < 0.01), and 51.31% (*p* < 0.05) at 60th, 90th, and 120th minutes, respectively (Figure 3(a)). The same figure revealed a significant reduction in the area under the curve (AUC) level of acarbose (*p* < 0.001) (Figure 3(b)). Similarly, the dose of 444 mg/kg of extract caused a significant reduction in blood glucose levels estimated at 58.50% (*p* < 0.01), 60.10% (*p* < 0.01), and 53.11% (*p* < 0.05) at the 60th, 90^th^, and 120th minute, respectively. A significant (*p* < 0.05) decrease in blood glucose levels of approximately 55.04% and 48.82% was also recorded with the dose of 222 mg/kg of extract at the 60th and 90th minute, respectively. The AUC level was significantly reduced at doses of 111 (*p* < 0.01), 222 (*p* < 0.001), and 444 mg/kg (*p* < 0.001) of extract (Figure 3(b)).

### 3.5. Effects of the Aqueous Extract of *C. polyantha* on Oral on Sucrose Tolerance Test

**Figure 4:**
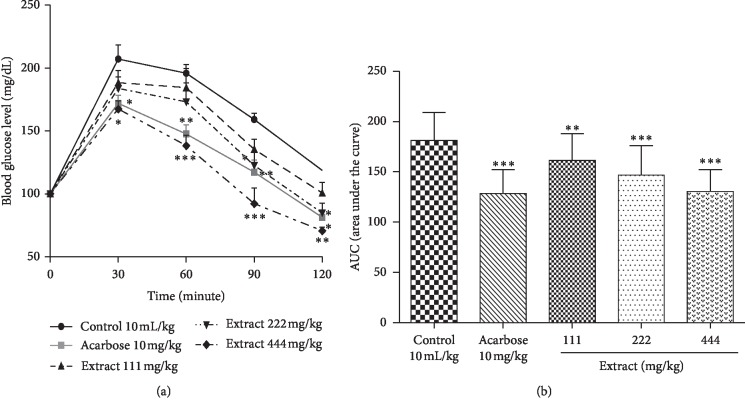
Effect of the aqueous extract of *Cissus polyantha* leafy stems on postprandial blood glucose level (a) and area under the curve (AUC) (b) after sucrose loading in normal rat. Values are expressed as mean ± SE of 5 rats in each group. ^*∗*^*p* < 0.05; ^*∗∗*^*p* < 0.01; ^*∗∗∗*^*p* < 0.001 compared to control. SE: standard error.

The results of the sucrose tolerance test are shown in Figures 4(a) and 4(b). As in the starch tolerance test, the curves representing the changes in blood glucose increased at the 30th minute and then gradually decreased until the 120th minute after the sucrose administration. However, throughout the observation period, the blood glucose levels of the control group remained higher than those of the acarbose-treated rats and the different doses of extract.

The regulation of postprandial glycaemia was greater in the rats given the reference product (acarbose) and in those treated with the extract at a dose of 444 mg/kg. In fact, acarbose resulted in a significant decrease in blood glucose of approximately 35.29% (*p* < 0.05), 48.21% (*p* < 0.01), 41.93% (*p* < 0.01), and 37.33% (*p* < 0.05) at the 30th, 60th, 90^th^, and 120th minutes, respectively. The extract (444 mg/kg) caused a significant drop in blood glucose of about 39.68% (*p* < 0.05), 57.61% (*p* < 0.001), 66.85% (*p* < 0.001), and 48.00% (*p* < 0.01) at the 30th, 60th, 90^th^, and 120th minutes, respectively. This decrease was less significant at a dose of 222 mg/kg of extract, i.e., approximately 36.56% and 33.93% at 60th and 90th minutes, respectively. However, the dose of 111 mg/kg had no effect on the hyperglycemia of the animals during the 2 hours of observation.

In addition, acarbose and the extract at a dose of 444 mg/kg significantly reduced (*p* < 0.001) the AUC level. Doses of 111 and 222 mg/kg of extract also resulted in a significant (*p* < 0.01, *p* < 0.05) reduction in the AUC level.

### 3.6. Effects of the Aqueous Extract of *C. polyantha* on Antioxidant Parameters

#### 3.6.1. Ferric-Reducing Antioxidant Power

In the iron-reducing test, ascorbic acid and extract showed a reducing capacity which increases with concentration. However, the effective concentration (EC_50_) of the reference substance (87.63 ± 3.22 *μ*g/mL) was largely higher than that of the extract (323.33 ± 2.46 *μ*g/mL) (Table 2).

Data are expressed as mean ± SE. EC_50_: effective concentration which corresponds to 0.5 of absorbance under the assayed conditions. SE: standard error.

#### 3.6.2. DPPH Antiradical Activity

The results of the DPPH antiradical activity of the extract and the positive control substance (BHA) revealed a concentration-dependent increase in percentage inhibition of free radical DPPH. It appears that BHA has a strong antiradical activity with an IC_50_ value of 80.88 *±* 1.33 *μ*g/mL, higher than the activity of the extract whose IC_50_ value was 393.25 *±* 0.65 *μ*g/mL (Table 3).

Data are expressed as mean ± SE. BHA: butylhydroxyanisole. IC_50_: inhibitor concentration to inhibit 50% of his activity under the assayed conditions. SE: standard error.

## 4. Discussion

*α*-Amylases and *α*-glucosidases are two enzymes involved in the digestion of carbohydrates, the main source of glucose in the body. Their inhibition may significantly reduce the increase in postprandial glucose level and may therefore be a therapeutic potential for the treatment of obesity and diabetes [2]. Some conventional drugs such as acarbose are used for this purpose. Unfortunately, they revealed various side effects and poor control of exacerbation [18]. However, natural inhibitors of digestive enzymes with lesser side effects have been discovered in various medicinal plants [19]. *Cissus polyantha* is one of those herbs traditionally used to treat diabetes. Phytochemical screening revealed that its leaves contain phenols, flavonoids, tannins, alkaloids, anthraquinones, steroids, carbohydrates, and glycosides [10]. The objective of this work was to determine the inhibitory properties of the aqueous extract of the leafy stems of *Cissus polyantha* on the activity of two digestive enzymes and to evaluate its antioxidant potential. Determination of phenols, flavonoids, and tannins indicated that these three metabolites are present in leafy stems of *Cissus polyantha*, in significant proportions. The leaves have been shown to contain quercetin flavonoids [20].

The in vitro inhibition tests of the *α*-amylase and *α*-glucosidase enzymes showed a strong concentration-dependent inhibitory activity of acarbose and *C. polyantha* extract. However, the extract exhibited the highest inhibition potential for *α*-glucosidase activity with an IC_50_ of 182.40 ± 0.54 *μ*g/mL compared to that of the *α*-amylase enzyme whose IC_50_ was of 216.14 ± 0.83 *μ*g/mL. Acarbose showed an overall inhibitory activity greater than that of the extract. These results have been confirmed by in vivo studies on starch and sucrose tolerance tests. During the starch tolerance test, the doses of 222 and 444 mg/kg of the extract produced a significant decrease in AUC (*p* < 0.001) and postprandial glycaemia at the 60th, 90^th^, and 120th minute. Concerning the sucrose tolerance test, a significant decrease in AUC and blood glucose level was noted after the 60th, 90^th^, and 120th minutes at the dose of 444 mg/kg of the extract. These results show that the aqueous extract of *C. polyantha* and acarbose delayed the digestion and absorption of starch and sucrose along the digestive tract, thus reducing postprandial hyperglycemia. From these results, it is observed that there is a good correlation between in vitro and in vivo tests. Acarbose contains compounds with structures similar to those of disaccharides and monosaccharides resulting from the digestion of carbohydrates, which allow them to bind to the *α*-glucosidase enzymes located on the brush border of the intestine, thus competitively inhibiting these enzymes [21]. According to Sales et al. [22], several phenolic compounds of the hydroxyl group confer an inhibitory activity of *α*-amylase and *α*-glucosidase. Quercetin and catechin act as competitive inhibitors of *α*-amylases [23]. By the same mechanism, the phenolic compounds contained in the aqueous extract of *C. polyantha* could inhibit the activity of the *α*-amylase and *α*-glucosidase enzymes, thereby decreasing postprandial hyperglycemia.

The study of the antioxidant power of the extract revealed a DPPH antiradical activity and a concentration-dependent iron-reducing potential. The antioxidant potential of a substance is attributed to its ability to break the chain of free radicals by giving a hydrogen atom [24]. In the antiradical test, the extract and the reference substance (BHA) showed an IC_50_ of the order of 393.25 ± 0.65 and 80.88 ± 1.33 g/mL, respectively. The antioxidant activity of a compound is inversely proportional to its IC_50_ value [25]. Mean effective concentrations (EC_50_) for iron reduction were 323.33 ± 2.46 g/mL for the extract and 87.63 ± 3.22 g/mL for ascorbic acid. The antioxidant activity of the extract was lower than that of the reference substances. These iron-reducing properties are attributed to phenolic compounds, especially flavonoids and tannins, whose hydroxyl groups can trap radical species [26] and chelate metal ions [27, 28]. The flavonoids present in the extract are therefore responsible for the antiradical activity observed. These results confirm those obtained by Pietta [29]. Free radicals and metal ions have been shown to cause lipid peroxidation, oxidative stress, and risk factor for type 2 diabetes [30]. It is evident that natural substances capable to inhibit polysaccharide digestion, intestinal absorption of glucose, and the prevention of oxidative stress, are potentially effective in preventing obesity and treating type 2 diabetes [31]. The leafy stems of *Cissus polyantha* would therefore be a source of these bioactive molecules.

## 5. Conclusion

The present study has shown that the aqueous extract of the leafy stems of *Cissus polyantha* has a remarkable antioxidant potential and inhibitory activity of the *α-amylase* and *α-glucosidase* enzymes. These effects would be due to its important phenolic composition whose quantitative study has revealed the varied presence of polyphenols, flavonoids, and tannins. These results could justify the use of *C. polyantha* leafy stems in traditional medicine for the treatment of type 2 diabetes and complications.

## Figures and Tables

**Table 1:** Quantitative analysis of some phytochemical substances present in leafy stem extracts of *Cissus polyantha.*

Secondary compounds	Total phenols (mgGAE/g dry wt.)	Total flavonoids (mg QE/g dry wt.)	Total tannins (mg CE/g dry wt.)
Extract	167.68 ± 0.6	7.54 ± 0.15	0.82 ± 0.5

**Table 2:** EC_50_ values calculated during ferric reducing antioxidant power.

	EC_50_ (*μ*g/mL)
Extract	323.33 *±* 2.46
Ascorbic acid	87.63 *±* 3.22

**Table 3:** IC_50_ values calculated during the DPPH antiradical activity.

	IC_50_ (*μ*g*/*mL)
Extract	393.25 *±* 0.65
BHA	80.88 *±* 1.33

## Data Availability

The data that support the findings of this study are available from the corresponding author upon reasonable request
